# Molecular Basis and Advances in Targeted Immunotherapy for Cancer

**DOI:** 10.3390/ijms24097802

**Published:** 2023-04-25

**Authors:** Antonio Macciò, Clelia Madeddu

**Affiliations:** 1 Department of Gynecologic Oncology, Unit of Obstetrics and Gynecology, ARNAS G. Brotzu, 09100 Cagliari, Italy; 2 Department of Surgical Sciences, University of Cagliari, 09100 Cagliari, Italy; 3 Department of Medical Sciences and Public Health, University of Cagliari, 09100 Cagliari, Italy; clelia_md@yahoo.it

Researchers have long attempted to stimulate the immune system of cancer patients as a therapeutic strategy. During these attempts, the mechanisms of immune escape have emerged and are now better defined, thus identifying new classes of immunotherapeutic drugs as immune checkpoint inhibitors (ICIs). ICI-based immunotherapy has dramatically improved the prognosis and survival of patients with advanced cancers. However, the identification of potential biomarkers useful to identify patients most likely to benefit from immunotherapy is, presently, still flawed.

The exact knowledge of the biological and molecular pathways involved in the regulation of immune response and immune escape is mandatory to understand the basis of modern immunotherapy, define the mechanisms involved in resistance, and develop new effective strategies.

Cancer cells can be considered pathogens; therefore, their related pathology depends on the extent of tumor growth and the effectiveness of immune responses. The immune response against cancer can be divided into two phases based on the mechanisms that define the body’s defense capacity. The initial phase is called resistance, in which the body tries to eliminate cancer cells, and the second is called tolerance, in which the body attempts to limit the health impacts caused by cancer. During the resistance phase, the immune system recognizes the antigenic diversity of the tumor and attempts to reduce the cancer cell burden once oncogenesis is initiated. Thus, the clinical manifestations of cancer highlight the low tumor immunogenicity and reduced ability of the immune system to control tumor cell growth through escape mechanisms [1].

Macrophages, dendritic cells, and T and B cells are the main contributors to antitumor resistance. Activation of the immune system, known as immunosurveillance, follows the classical steps of antigen recognition, antigen presentation to effector and regulatory T cells, and the physiological end of the immune response through immune checkpoint pathways. Macrophages are essential during the initial phase of the immune response because of their antineoplastic properties, capacity to synthesize cytokines, and interactions with and recruitment of helper cells and cytotoxic T lymphocytes. However, their persistent activation leads to the impairment of effective T cell responses by causing T cell exhaustion, a condition in which lymphocytes, even when activated, are nonfunctional and subsequently undergo programmed cell death, which may contribute to immunodepression and cancer immune escape. Therefore, the growth of a tumor that overcomes resistance mechanisms underlines the lack of efficacy of a specific immune response, which is followed by a macrophage-mediated chronic inflammatory response with related symptoms, the persistence of which leads to tolerance. The tolerance phase is then characterized by several symptoms, such as anemia, anorexia, and muscle wasting, which may compromise the efficacy of the immune response and immunotherapy [1].

Therefore, the immune profile and composition of the tumor microenvironment (TME) may play a crucial role in influencing the antitumor immune response and efficacy to immunotherapy. Considering the importance of both tumor and TME immune subtypes in immunotherapy, Lai et al. [2] proposed a predictive pathway-related biomarker associated with immune response. They constructed an allograft rejection (AR)-related five-gene signature pathway that could independently predict the prognosis of lower-grade gliomas (LGG) and response to ICIs. They found that patients with high-AR LGG had higher tumor mutation burden (TMB), immunophenotype score (IPS), IMmuno-PREdictive Score (IMPRES), T-cell-inflamed gene expression profile (GEP) score, and MHC I association immunoscore (MIAS). Moreover, they found that CD8+ T cells, M1 and M2 macrophages, and eosinophils were differentially distributed between the high- and low-AR groups, indicating a potential association between the signature and the tumor immune microenvironment. The results revealed that patients with LGG may show a different response to immunotherapy according to the proposed risk stratification, and that patients with high-AR LGG were more likely to respond to PD-1 blockade therapy.

Tumor-derived extracellular vesicles (EVs) are among the tumor-related factors that contribute to the establishment of an immunosuppressive TME. These particles contribute to tumor immune escape in several ways: (a) by suppressing CD8+ T cell activation and proliferation; (b) by expressing ligands that bind to T cell death receptors, such as the Fas ligand; (c) by polarizing macrophages toward the M2 phenotype; and (d) by inducing the secretion of inflammatory cytokines. Moreover, EVs express checkpoint molecules on their surface, allowing tumor cells to evade immune cell attack and acquire resistance to ICIs. Additionally, tumor-derived EVs interact with lymph-node-resident lymphatic endothelial cells and induce the expression of PD-L1 on their surface, thus inducing T cell apoptosis and resulting in tumor immune escape and progression. In their review, Kiya et al. [3] discussed the association between extracellular vesicles and tumor progression via the immune system, as well as the clinical application of EVs as biomarkers and therapeutic agents.

Starting with an analysis of the unique immune environment of the liver and subsequent alterations to the TME, a review by Ruff et al. [4] discusses the currently approved and most emerging immunotherapy approaches tested both in preclinical research and clinical trials, focusing on the representative setting of hepatocellular carcinoma and biliary tract carcinoma, which represent a recent and promising area of investigation of immunotherapy, although with controversial clinical results.

Understanding the mechanisms of resistance to ICIs and identifying parameters to predict optimal response to treatment are cornerstones of modern immuno-oncology research. The discovery of predictive biomarkers for ICI-based immunotherapy is evolving. Programmed death ligand 1 (PD-L1) expression in tumor samples is recognized as a marker of eligibility for immunotherapy, although not univocally. Mutations in other genes, particularly those involved in mismatch repair (MMR), may predict the efficacy of immunotherapy. Mutations in MMR genes impair DNA repair, leading to microsatellite instability associated with neoantigen formation. Mutations in genes involved in DNA repair and replication have been associated with a higher mutational load and correlated with an increased response to PD-1 blockade. However, in some cases, specific mutations have been associated with immunotherapy resistance, although the underlying mechanisms remain controversial and require further investigation. For example, current evidence suggests that immunotherapy is not beneficial for patients with non-small-cell lung cancer (NSCLC) carrying EGFR mutations. Indeed, EGFR mutations have been shown to be associated with immunosuppressive TME, lower TMB, and increased PD-L1 expression, which may lead to a poor response to ICIs [5]. Madeddu et al. [5] discussed the role of EGFR mutations in influencing the TME and immune response, as well as resistance to immunotherapy, dynamic changes in the TME and immune cells during tyrosine kinase inhibitor (TKI) treatment, strategies for overcoming resistance to immunotherapy, and the rationale for combining TKI treatment and immunotherapy in EGFR-mutated NSCLC. As discussed in this review, evidence from the literature supports the notion that the TME in EGFR-mutated NSLC is immunosuppressive, with reduced TMB, the low expression of PD-L1, low TIL numbers, and high Treg infiltration. Additionally, NSCLC harboring EGFR mutations typically present with a non-inflamed TME, which has been associated with a poor response to immunotherapy. In this regard, it remains unclear whether there are differences in the TME and ICI efficacy between NSCLC with different EGFR mutation subtypes. Several potential approaches to improve the response to immunotherapy in EGFR-mutated NSCLC have been tested. Targeting TAM and DC therapy may be an interesting future direction for patients with EGFR mutations. In addition, combining ICI with TKI may be another effective therapeutic strategy.

Moreover, next-generation sequencing (NGS) may be fundamental in identifying predictive biomarkers of response/resistance to ICIs as a prelude to the development of rational treatment strategies, especially in pre-treated patients. Grenda et al. [6] described a characteristic case of a patient with metastatic squamous NSCLC who progressed after a few cycles of immunotherapy, in which the decision to perform NGS was crucial for the prediction of the NSCLC course and treatment choice. In this case, mutations in two genes, the *BRAF* oncogene and the *NF1* tumor suppressor gene, indicated the use of targeted therapy with dabrafenib and trametinib, which achieved disease stabilization. This article demonstrates how the presence of coexisting mutations in these genes affects the disease course and immunotherapy efficacy and is crucial in the planning of treatment.

Novel immunotherapeutic approaches, including the immune modulation of the TME by combining the targeted inhibition of different immune mediators and tumor proliferative pathways, could represent a potentially effective strategy in the future. Jacques et al. [7] reported a preclinical exploratory study that tested a sequential micro-immunotherapy medicine, MIM-seq, for its immunomodulatory effects on human primary M1 and M2 macrophages and its antiproliferative effects on in vitro and in vivo models of colorectal cancer (CRC). Their data suggested that MIM-seq has antitumor properties against CRC and an immunomodulatory effect towards the mediators of inflammation, whose systemic dysregulation is considered a poor prognosis for patients.

In any case, when considering the mechanisms that modulate the effectiveness of immunotherapy, the evaluation of the patient’s general status is crucial. In particular, a compromised nutritional status, especially in the context of cancer cachexia, which is a complex metabolic syndrome driven by cancer-related chronic inflammation typical of advanced cancer patients, may affect the immune response and impair immunotherapy efficacy. In this regard, a recent prospective study by our group supports the evidence that cachexia, with its related changes in inflammatory, body composition, energy metabolism, and nutritional parameters, is a key prognostic and predictive factor for ICI-based immunotherapy in patients with advanced NSCLC [8]. Consistently, blocking inflammation and related nutritional and metabolic derangements with specific drugs may be a rational strategy to improve immunotherapy efficacy in advanced cancer patients [9].

In conclusion, taking the concepts mentioned above as references, we can affirm that the basis for immunotherapy and immunomodulation should require clinicians to verify: (i) the presence of an immunogenic antigen, (ii) the activation of T cells, as indicated by the increased expression of immune checkpoints (such as CTLA4 and PD-1), (iii) the integrity of effector T cell functions, (iv) the absence of an immunosuppressive TME characterized by the presence of Treg cells, immunosuppressive macrophage populations, PD-L1 expression, or cytokines with immunosuppressive actions, (v) the presence of molecular and genetic alterations that can influence the immune response, the TME profile, and the efficacy of immunotherapy, and (vi) the absence of a chronic inflammatory status typical of the tolerance phase, associated with oxidative stress, with consequent changes in energy metabolism and systemic debilitating symptoms [9].

Our Special Issue, with three original articles and three reviews, contributes to the discussion and clarification of these issues and highlights how understanding the precise role of the immune response in specific subsets of patients in relation to the stage of disease, tumor type, molecular profile, TME composition, and general patient status is a goal that should be pursued vigorously. This merged evidence provides new insights for a more personalized and potentially effective application of immunotherapy in clinical practice.
